# Comparison of Two Devices and Two Breathing Patterns for Exhaled Breath Condensate Sampling

**DOI:** 10.1371/journal.pone.0027467

**Published:** 2011-11-07

**Authors:** Eva-Maria Hüttmann, Timm Greulich, Akira Hattesohl, Severin Schmid, Sarah Noeske, Christian Herr, Gerrit John, Rudolf A. Jörres, Bernd Müller, Claus Vogelmeier, Andreas Rembert Koczulla

**Affiliations:** 1 Division for Pulmonary Diseases, Department of Internal Medicine, Philipps-University Marburg, Marburg, Germany; 2 Comprehensive Pneumology Center, Institute of Lung Biology and Disease, Helmholtz Zentrum Munich, Neuherberg, Germany; 3 Institute and Outpatient Clinic for Occupational, Social and Environmental Medicine, Ludwig-Maximilians-University, Munich, Germany; University of Pittsburgh, United States of America

## Abstract

**Introduction:**

Analysis of exhaled breath condensate (EBC) is a noninvasive method to access the epithelial lining fluid of the lungs. Due to standardization problems the method has not entered clinical practice. The aim of the study was to assess the comparability for two commercially available devices in healthy controls. In addition, we assessed different breathing patterns in healthy controls with protein markers to analyze the source of the EBC.

**Methods:**

EBC was collected from ten subjects using the RTube and ECoScreen Turbo in a randomized crossover design, twice with every device - once in tidal breathing and once in hyperventilation. EBC conductivity, pH, surfactant protein A, Clara cell secretory protein and total protein were assessed. Bland-Altman plots were constructed to display the influence of different devices or breathing patterns and the intra-class correlation coefficient (ICC) was calculated. The volatile organic compound profile was measured using the electronic nose Cyranose 320. For the analysis of these data, the linear discriminant analysis, the Mahalanobis distances and the cross-validation values (CVV) were calculated.

**Results:**

Neither the device nor the breathing pattern significantly altered EBC pH or conductivity. ICCs ranged from 0.61 to 0.92 demonstrating moderate to very good agreement. Protein measurements were greatly influenced by breathing pattern, the device used, and the way in which the results were reported. The electronic nose could distinguish between different breathing patterns and devices, resulting in Mahalanobis distances greater than 2 and CVVs ranging from 64% to 87%.

**Conclusion:**

EBC pH and (to a lesser extent) EBC conductivity are stable parameters that are not influenced by either the device or the breathing patterns. Protein measurements remain uncertain due to problems of standardization. We conclude that the influence of the breathing maneuver translates into the necessity to keep the volume of ventilated air constant in further studies.

## Introduction

Exhaled breath condensate (EBC) is a relatively new and completely noninvasive method to access the epithelial lining fluid of the lungs [1]. It should be particularly useful in longitudinal studies and questions requiring repeated measurements. In contrast to clinically established methods like bronchoalveolar lavage (BAL) sampling, EBC can be performed without any problems because it is a safe and simple procedure even in small children [2].

In the past years there have been numerous studies using EBC to analyze inflammatory diseases, examining unspecific markers like conductivity or pH on the one hand [3]–[5], but also looking at very specific inflammatory cytokines like interleukin (IL)-1β, IL-4, IL-6 and IL-10 on the other hand [6]–[10]. However, so far the reported mediator levels vary greatly, enabling studies on EBC to make relative statements only. This is due to a lack of work on standardization and sampling technique that might have an influence on observed results. There has been an abundance of different condensing equipment, but nowadays the commercially available devices RTube (Respiratory Research Inc., VA) and ECoScreen (VIASYS Healthcare, Hoechberg, Germany) are used in most studies. As differences in the condensing materials, cooling temperature and air trapping exist, there is a need for information on how these differences influence volume and composition of the EBC.

Two studies found differences in amounts of proteins such as eotaxin and cysteinyl-leukotriene [11] as well as in pH levels [12]. In contrast, in our comparison of ECoScreen I and RTube the EBC pH values were not significantly different for the two devices [13]. Variation of the coating material also seemed to have an impact on the amounts of albumin and 8-isoprostane [14]. In another study, Czebe et al. compared three devices and different cooling temperatures for the RTube but did not find significant differences in volume and total protein in samples collected with the RTube and ECoScreen. While in some studies different cooling temperatures seemed to have an influence on pH values, the largest study addressing this issue demonstrated that the cooling temperature had no significant impact on EBC [15]. The volume of EBC was reported to be higher in samples collected using the ECoScreen [16].

Until now, there have been few data regarding the influence of different breathing maneuvers on composition and volume of the EBC. Patients who breathe either performing tidal breathing or forced expiration (hyperventilation) might ventilate different areas of the lung leaving the researcher with only a vague idea of the origin of the condensed breath. Furthermore, the fact that the ECoScreen I can not be purchased anymore is another major reason for the comparison of ECoScreen Turbo and RTube. We wanted to address the question as to what extend breathing patterns and different collection devices influence a variety of non-specific markers in EBC.

## Methods

### Study participants

EBC was collected from 10 healthy non-smoking controls at the age of 24.8 years ±2.78 years (23 years to 30 years) that showed no clinical sign of inflammation at the time of their measurements. All patients underwent a single study visit during which EBC was collected four times in a crossover design, twice with every device; once with every device while performing hyperventilation and once with tidal breathing. All participants gave written informed consent and the study (no. 59/06) as well as the informed consent form was approved by the ethics committee of the Philipps University Marburg, Germany.

### EBC Collection

EBC samples were collected during 10 minutes of quiet breathing and 10 minutes of hyperventilation through a single-use disposable RTube collector (Respiratory Research, Inc.; Charlottesville, VA) and in a crossover design with the ECoScreen Turbo (VIASYS Healthcare GmbH, Hoechberg, Germany) also once during quiet breathing (again for 10 minutes) and during 10 minutes of hyperventilation while subjects were wearing a nose clip. The aluminum sleeve of the device had been cooled to an initial temperature of −20°C prior to collection [15], [16].

The ECoScreen device is equipped with the ECoVent for recording of the exhaled breath volume and constantly cooled to approximately −4°C (VIASYS Healthcare GmbH, Hoechberg, Germany).

### pH and Conductivity

For pH determination, 250 µl of EBC were transferred into a polyethylene tube. Samples were de-aerated with a gentle argon flow (Linde Gas, Germany, purity 99.9%) for at least 20 min as described before until pH readings were stable. pH was measured with a glass electrode [13], [17]. Conductivity measurements were performed in 100 µl EBC using a glass microcell (LDM/S; WTW, Weilheim, Germany) at a temperature of 25°C as described before [17].

### Protein Sampling

Total protein amount was measured using spectroscopy according to the manufacturer's guidelines (Spectrophotometer, NanoDrop 1000, Peqlab Biotechnologie GmbH, Erlangen, Germany).

### ELISA of CCP and SP-A

Concentrations of human Clara cell secretory protein (CCP) and surfactant protein A (SP-A) in EBC were determined by commercially available kits for enzyme-linked immunosorbent assay (ELISA, BioVendor, Heidelberg, Germany). For ELISA of CCP, 1 ml of EBC was lyophilized using a speed vac (Bachofer, Reutlingen, Germany) and the sample was resuspended in a volume of 100 µl. For SP-A ELISA, 100 µl of native EBC was used. Absorption at 450 nm was detected using a Tecan Ultra 384 Reader (Tecan, Crailsheim, Germany).

### Western blot of CCP and SP-A

For Western blots of CCP and SP-A, EBC samples were mixed with loading buffer, boiled for 1.5 min, separated by polyacrylamide gel electrophoresis (PAGE) on 8% tris-tricine sodium dodecyl sulfate (SDS) gels (Invitrogen), and transferred onto nitrocellulose membranes. Membranes were blocked with 5% non-fat dry milk in phosphate buffered saline (PBS) and probed with polyclonal antibodies against CCP (BioVendor, Heidelberg, Germany) and SP-A (Santa Cruz Biotechnology, Santa Cruz, CA) at 4°C overnight. Specific bands were visualized using horseradish peroxidase (HRP)-conjugated secondary antibodies and enhanced chemiluminescence (Pierce, Rockford, IL) and developed onto x-ray films (Pierce).

### Electronic Nose

For both experiments the Cyranose 320 (C-320, Smiths Detection Group Ltd., Watford, UK) was used. This is a hand-held device capable of detecting so-called smellprints by analyzing mixtures of volatile organic compounds (VOCs). The C-320 is equipped with 32 chemical sensors that respond differently to different mixtures of VOCs. The sensors consist of conducting chemiresistors made from carbon black nanocomposites that change their resistance in response to VOCs.

One measurement with the C-320 included three consecutive steps:

1. Baseline: The sensors were exposed to reference air.
2. Sampling: The sensors were exposed to sample air. The changes of sensor resistances compared to reference air were recorded.
3. Purging: The sensors were refreshed by exposing them to ambient air.

For the measurement with the electronic nose 200 µl of the collected EBC was heated up to 37°C and gently bubbled with argon gas for two minutes to decarbonate it and to increase the gas phase. Ambient air was uses as reference air while baselining for ten seconds. The snout of the C-320 was hold above the surface drawing a sample for ten seconds.

### Data Analysis

We conducted a formal power calculation for the pH using previously published data [13] and found that with alpha = 0.05, a power = 0.8 and a minimum EBC pH difference of 0.193 we needed to include 10 subjects in each group.

Statistical analysis was performed using SigmaStat, MedCalc 11.1.1.0 and GraphPad Prism 5.0. Data are presented as mean ± standard error of the mean (SEM). D'Agostino and Pearson's omnibus normality test was performed to test for normal distribution. Normally distributed values were compared using the paired Student's *t*-test. If the normality test failed, the Wilcoxon signed-rank test was employed. Differences between values of groups were explored by one-way analysis of variance (ANOVA) followed by *post-hoc* multiple comparisons according to Tukey's test. Intraclass correlation analysis (ICC) was performed for each group to estimate the reliability of single measurements.

The electronic nose data were analyzed using the classifier linear discriminant analysis (LDA). EBC data were preprocessed by centering and normalizing. Additionally the Mahalanobis distances (MDs) between the groups and a 100-fold cross-validation using 10% of the data as test data was performed to calculate the cross-validation value (CVV).

## Results

### Study Population

We included 10 healthy controls (4 male, 6 female) with a mean age of 24.8 years±2.78 years (23 years to 30 years) a mean BMI of 21.52 kg/m^2^±0.72 kg/m^2^ (17.5 kg/m^2^ to 25.9 kg/m^2^). All were non-smokers and had no clinical signs of acute or chronic inflammation at the time of our measurements. Baseline characteristics are displayed in table 1.

**Table 1:** Baseline Characteristics.

Number	10
Male/female	4/6
Age [years]	24.8±2.78 (23–30)
BMI [kg/m^2^]	21.52±0.72 (18–)
Pack years	0

### Volume

**Figure 1:**
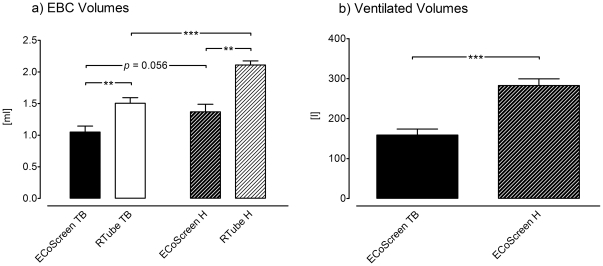
The figure shows the sample volume of exhaled breath condensate (EBC) after ten minutes collection. **a**. After tidal breathing (TB) and hyperventilation (TB) EBC volumes with RTube were higher compared with ECoScreen turbo (*p*<0.001) Hyperventilation causes higher EBC volumes compared with tidal breathing in both devices (*p*<0.0001 in RTube, *p* = 0.056 in ECoScreen). b. Hyperventilation via the ECoScreen turbo caused a 1.78 fold higher movement of ventilated air (*p*<0.0001).

Comparing our two devices with tidal breathing (TB) manoeuvres the RTube yielded significantly higher volumes of EBC than the ECoScreen (RTube: 1.51 ml±0.09 ml vs. ECoScreen: 1.05 ml±0.09 ml, *p*<0.001; figure 1a, left two bars). Performing hyperventilation (H) the RTube yielded higher volumes of EBC than the ECoScreen (RTube: 2.11 ml±0.07 ml vs. ECoScreen: 1.37 ml±0.12 ml, *p*<0.001; figure 1a, right two bars).

Within the same device, hyperventilation yielded higher volumes of EBC than tidal breathing: Using the RTube we obtained 2.11 ml±0.07 ml with hyperventilation and 1.51 ml±0.09 ml with tidal breathing (*p*<0.0001; figure 1a, white bars) and using the ECoScreen we obtained 1.37 ml±0.12 ml with hyperventilation and 1.05 ml±0.09 ml with tidal breathing although this difference did not reach statistical significance (*p* = 0.056; figure 1a, grey bars).

Taken together, the RTube yielded higher EBC volumes than the ECoScreen, and the hyperventilation higher EBC volumes than tidal breathing.

Regarding the ventilated volume of air, as expected, hyperventilating participants moved a higher volume of air than patients performing tidal breathing using the ECoScreen Turbo (H: 282.9 l±16.49 l vs. TB: 158.82 l±15.05 l, *p*<0.0001; figure 1b). Because the volume of ventilated air was not measured using the RTube we could not perform this measurement in this device.

### pH

**Figure 2:**
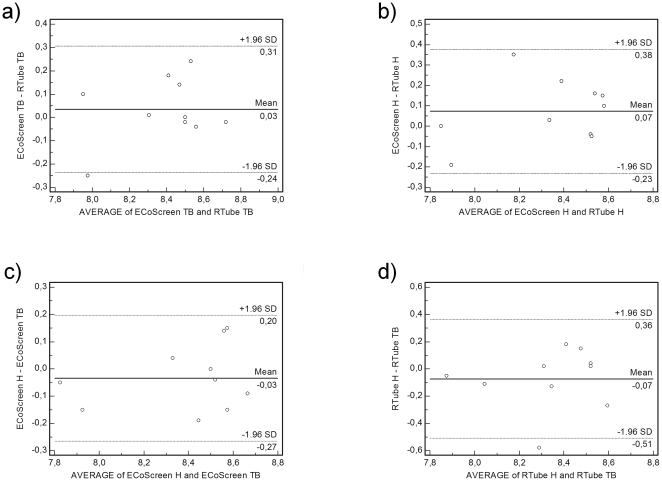
Bland Altman Plots are shown to display differences in individual measurements of the exhaled breath condensate (EBC) under certain conditions. Neither the device nor the ventilation pattern changed the EBC pH significantly. a. RTube and ECoScreen showed comparable pH values in EBC in tidal breathing (TB). b. RTube and ECoScreen showed comparable pH values in EBC in hyperventilation (H). c. The breathing manoeuvres did not produce significant differences in EBC pH using the ECoScreen. d. The breathing manoeuvres did not produce significant differences in EBC pH using the RTube.

In tidal breathing RTube and ECoScreen resulted in comparable pH values (RTube: 8.38±0.08 vs. ECoScreen: 8.41±0.09, *p* = 0.46). The calculation of the ICC yielded 0.86 (95% confidence interval (CI) 0.55 to 0.96) with lower and upper limits of agreement of −0.24 (95% CI −0.41 to −0.06) and 0.31 (95% CI 0.13 to 0.48; figure 2a). Performing hyperventilation we also obtained values in good agreement (RTube: 8.3±0.08 vs. ECoScreen: 8.38±0.1, *p* = 0.17) with an ICC of 0.84 (95% CI 0.49 to 0.96) and limits of agreement of −0.23 (95% CI −0.43 to −0.03) and 0.37 (95% CI 0.18 to 0.57; figure 2b).

Testing for differences regarding the ventilatory maneuvers in one of the devices we did not detect significant differences. Using the ECoScreen we obtained very good agreement (TB: 8.41±0.09 vs. H: 8.38±0.1, *p* = 0.39) with an ICC of 0.92 (95% CI 0.73 to 0.98). The limits of agreement were −0.27 (95% CI −0.42 to −0.12) and 0.2 (95% CI 0.05 to 0.35; figure 2c). Using the RTube the data showed moderate agreement (TB: 8.38±0.08 vs. H: 8.3±0.08, *p* = 0.33) with an ICC of 0.61 (95% CI 0.04 to 0.88). The lower limit of agreement was −0.51 (95% CI −0.79 to −0.23) and the upper limit of agreement was 0.36 (95% CI 0.08 to 0.64; figure 2d).

We were able to show that neither the device nor the ventilatory maneuver changed the obtained pH significantly. The intraclass correlation coefficients testing for comparability between devices were 0.84 and 0.86, meaning good agreement. The ICCs testing for comparability between different breathing maneuvers were 0.61 and 0.92, indicating moderate to very good alignment.

### Conductivity

**Figure 3:**
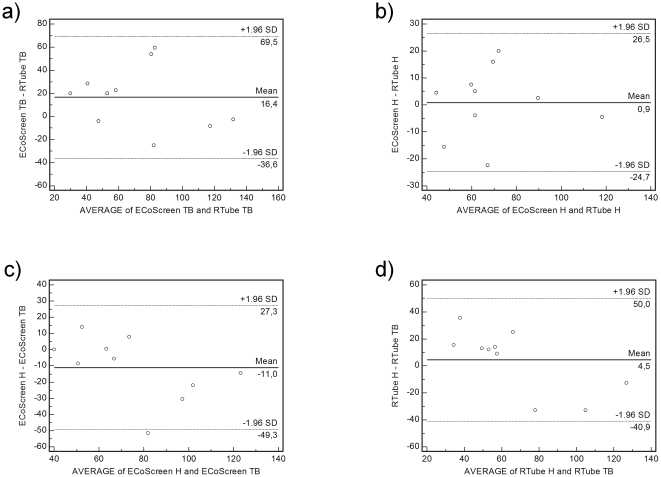
Bland Altman Plots are shown comparing the conductivity of exhaled breath condensate under different conditions. Neither the device nor the breathing pattern changed the conductivity of the EBC significantly. a. In tidal breathing (TB) RTube and ECoScreen did not produce statistically different conductivity values. b. RTube and ECoScreen did not produce statistically different conductivity values collecting EBC with a hyperventilation (H) maneuver. c. The breathing manoeuvres did not produce significant differences in EBC conductivity using the ECoScreen. d. The breathing manoeuvres did not produce significant differences in EBC conductivity using the RTube.

In tidal breathing RTube and ECoScreen did not result in statistically different values (RTube: 64.15 µS/cm±12.25 µS/cm vs. ECoScreen 80.6 µS/cm±10.21 µS/cm, *p* = 0.087), the ICC was 0.66 (95% CI 0.13 to 0.9) with a lower limit of agreement of −36.58 (95% CI −70.84 to −2.31) and an upper limit of agreement of 69.48 (95% CI 35.21 to 103.74; figure 3a). Performing hyperventilation we found concordant values (RTube: 68.7 µS/cm±7.02 µS/cm vs. ECoScreen 69.6 µS/cm±7.14 µS/cm, *p* = 0.83) with an ICC of 0.84 (95% CI 0.48 to 0.96). The lower and upper limits of agreement were −24.73 (95% CI −41.28 to −8.17) and 26.53 (95% CI 9.97 to 43.08; figure 3b).

Testing for differences regarding the ventilatory maneuvers in one of the devices the maneuvers did not result in significant differences. On average, the data showed good agreement using the RTube (TB: 64.15 µS/cm±12.25 µS/cm vs. H: 68.7 µS/cm±7.02 µS/cm, *p* = 0.55) with an ICC of 0.74 (95% CI 0.26 to 0.93). The lower and upper limits of agreement were −40.92 (95% CI −70.29 to −11.54) and 50.01 (95% CI 20.64 to 79.39; figure 3c), indicating high differences for some individuals. Using the ECoScreen, there was also no statistically significant difference (TB: 80.6 µS/cm±10.21 µS/cm vs. H: 69.6 µS/cm±7.14 µS/cm, *p* = 0.11). The ICC was 0.72 (95% CI 0.23 to 0.92) and the limits of agreement were −49.26 (95% CI −73.98 to −24.54) and 27.26 (95% CI 2.54 to 51.98; figure 3d), again indicating rather high differences for individual measurements.

In summary, we demonstrated that neither the device nor the breathing pattern changed the measured conductivity significantly. The intraclass correlation coefficients demonstrated moderate to good agreement (0.66 to 0.84). However, the wide limits of agreement demonstrate poor concordance regarding different devices in tidal breathing and different ventilatory maneuvers in the ECoScreen.

### Protein Measurements

**Figure 4:**
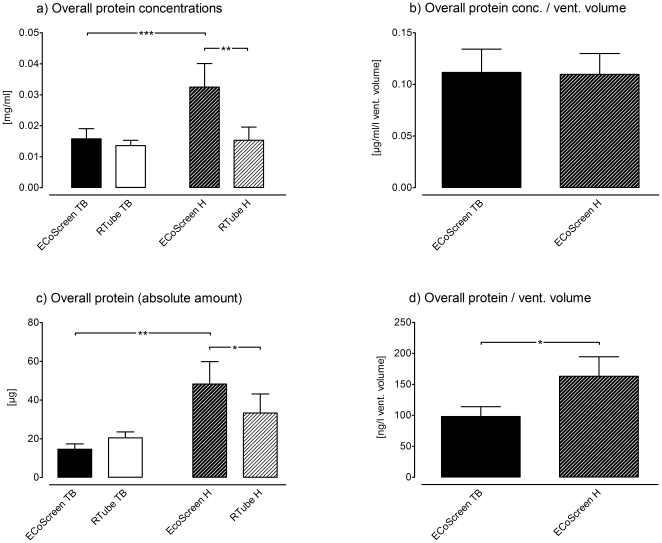
Displayed are the overall protein measurements in four different ways. a. Comparing the ECoScreen and RTube EBC protein concentration after tidal breathing (TB) no statistical significant difference could be shown (*p* = 0.51). After hyperventilation (H) ECoScreen resulted in higher protein concentrations than the RTube (*p*<0.001). Comparing the two manoeuvres, hyperventilation yielded higher concentrations than tidal breathing, but this difference was significant only in the ECoScreen (*p*<0.0001). b. To the volume of ventilated air normalized protein concentrations in EBC collected by the ECoScreen device did not show a difference between tidal breathing and hyperventilation (*p* = 1). c. Analyzing the total protein amount in EBC, hyperventilation with ECoScreen resulted in higher protein values compared to RTube (*p*<0.05). Comparing hyperventilation with tidal breathing in the ECoScreen device, hyperventilation resulted in higher absolute protein amounts (*p*<0.001). d. By normalizing the absolute protein amount in EBC to the volume of ventilated air using the ECoScreen turbo hyperventilation expressed higher overall protein values/ventilated volume (*p*<0.05).Within the same device hyperventilation yielded higher overall protein concentrations of EBC than tidal breathing, though the difference was statistically significant only in the ECoScreen device (H: 0.033 mg/ml±0.008 mg/ml vs. TB: 0.016 mg/ml±0.003 mg/ml; *p*<0.05; figure 4a, grey bars).

- **Overall Protein.** Comparing the two different devices performing tidal breathing maneuvers the ECoScreen yielded higher protein concentrations in EBC than the RTube (ECoScreen: 0.016 mg/ml±0.003 mg/ml vs. RTube: 0.014 mg/ml±0.002 mg/ml, *p* = 0.51; figure 4a, left two bars), although the difference was not statistically significant. When performing hyperventilation the ECoScreen resulted in significantly higher protein concentrations than the RTube (ECoScreen: 0.033 mg/ml±0.008 mg/ml vs. RTube: 0.015 mg/ml±0.004 mg/ml, *p*<0.001; figure 4a, right two bars).

When the overall protein concentrations were normalized to the volume of ventilated air (ECoScreen Turbo only), there was no difference detectable (H: 0.11 µg/ml/l±0.02 µg/ml/l vs. TB: 0.11 µg/ml/l±0.02 µg/ml/l; *p* = 1; figure 4b).

When we plotted the total protein amount instead of the protein concentration (thus, we multiplied the concentration with the volume of obtained EBC), the results remained rather stable. In tidal breathing the devices did not differ regarding the total protein amount (ECoScreen: 14.61 µg±2.78 µg vs. RTube: 20.47 µg±3.07 µg; *p* = 0.18; figure 4c, left bars). In hyperventilation the ECoScreen resulted in higher absolute protein amounts than the RTube (ECoScreen: 48.31 µg±11.54 µg vs. RTube 33.33 µg±9.85 µg; *p*<0.05; figure 4c, right bars). Within the same device, hyperventilation yielded higher absolute amounts than tidal breathing though the difference was significant only in the ECoScreen (H: 48.31 µg±11.54 µg vs. TB: 14.61 µg±2.78 µg; *p*<0.01; figure 4c, grey bars).

Finally, we normalized the absolute amount to the volume of ventilated air (ECoScreen Turbo only). With this method the absolute protein amount per liter of ventilated air was higher in hyperventilation compared to tidal breathing (H: 163.1 ng/l±31.44 ng/l vs. TB: 98.16 ng/l±15.79 ng/l; *p*<0.05; figure 4d).

**Figure 5:**
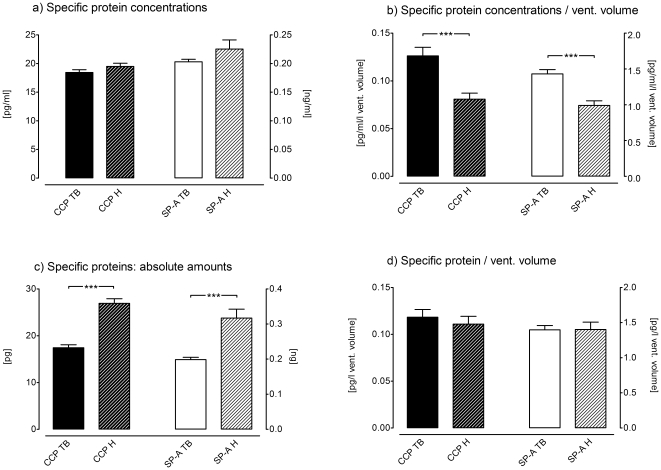
Specific protein measurements are displayed in four different ways. a. The breathing manoeuvres tidal breathing (TB) and hyperventilation (H) and also the devices RTube and ECoScreen turbo had no effect on the total concentration of Clara cell protein (CCP) and surfactant protein-A (SP-A), respectively (*p* = 0.17; *p* = 0.16). b. Normalizing the CCP and SP-A protein concentrations to ventilated volume revealed lower CCP and SP-A values under hyperventilation conditions (*p*<0.001; *p*<0.0001). c. Absolute amount of CCP and SP-A. Hyperventilation leads to significant higher SP-A and CCP levels (*p*<0.0001 for both). d. Normalizing the absolute amount of SP-A and CCP to the volume of ventilated air resulted in no significant difference of CCP and SP-A levels comparing hyperventilation with tidal breathing.

- **Specific Protein Measurements.** Using the ECoScreen we compared the concentration of CCP after tidal breathing and after hyperventilation and did not find significant differences. The CCP concentration after hyperventilation was 19.49 pg/ml±0.57 pg/ml compared to 18.44 pg/ml±0.48 pg/ml (*p* = 0.17; figure 5a, left bars). Comparably, the concentration of SP-A did not differ significantly with regard to the ventilatory maneuver (TB: 0.203 ng/ml±0.004 ng/ml vs. H: 0.225 ng/ml±0.016 ng/ml; *p* = 0.16; figure 5a, right bars).

When normalizing the concentration of SP-A and CCP to the volume of ventilated air, the specific protein measurements now revealed significantly higher protein concentrations per volume of ventilated air obtained after tidal breathing compared to hyperventilation regarding CCP (TB: 0.13 pg/ml/l±0.009 pg/ml/l vs. H: 0.08 pg/ml/l±0.006 pg/ml/l; *p* = 0.0003; figure 5b, left bars) and regarding SP-A (TB: 1.43 pg/ml/l±0.06 pg/ml/l vs. H: 0.99 pg/ml/l±0.06 pg/ml/l; *p*<0.0001; figure 5b, right bars).

Plotting the absolute amounts instead of the concentrations we found different results. Now, hyperventilation seemed to yield higher absolute amounts of specific proteins compared to tidal breathing. This was true for CCP (H: 26.92 pg±0.99 pg vs. TB: 17.43 pg±0.63 pg; *p*<0.0001; figure 5c, left bars) and for SP-A (H: 0.32 ng±0.025 ng vs. TB: 0.19 ng±0.006 ng; *p*<0.0001; figure 5c, right bars).

After normalizing the absolute amount of SP-A and CCP to the volume of ventilated air, the results changed again. In this setting the ventilatory maneuver had no influence on the amount per volume of ventilated air, neither regarding CCP (H: 0.11 pg/l ± 0.008 pg/l vs. TB: 0.12 pg/l±0.008 pg/l; *p* = 0.23; figure 5d, left bars) nor regarding SP-A (H: 1.4 pg/l±0.1 pg/l vs. TB: 1.39 pg/l±0.06 pg/l; *p* = 0.96; figure 5d, right bars).

To summarize the protein measurements: The reported results changed dramatically when reported differently (concentration, concentration per liter ventilated volume, absolute amount, amount per liter ventilated volume).

### Electronic Nose

**Figure 6:**
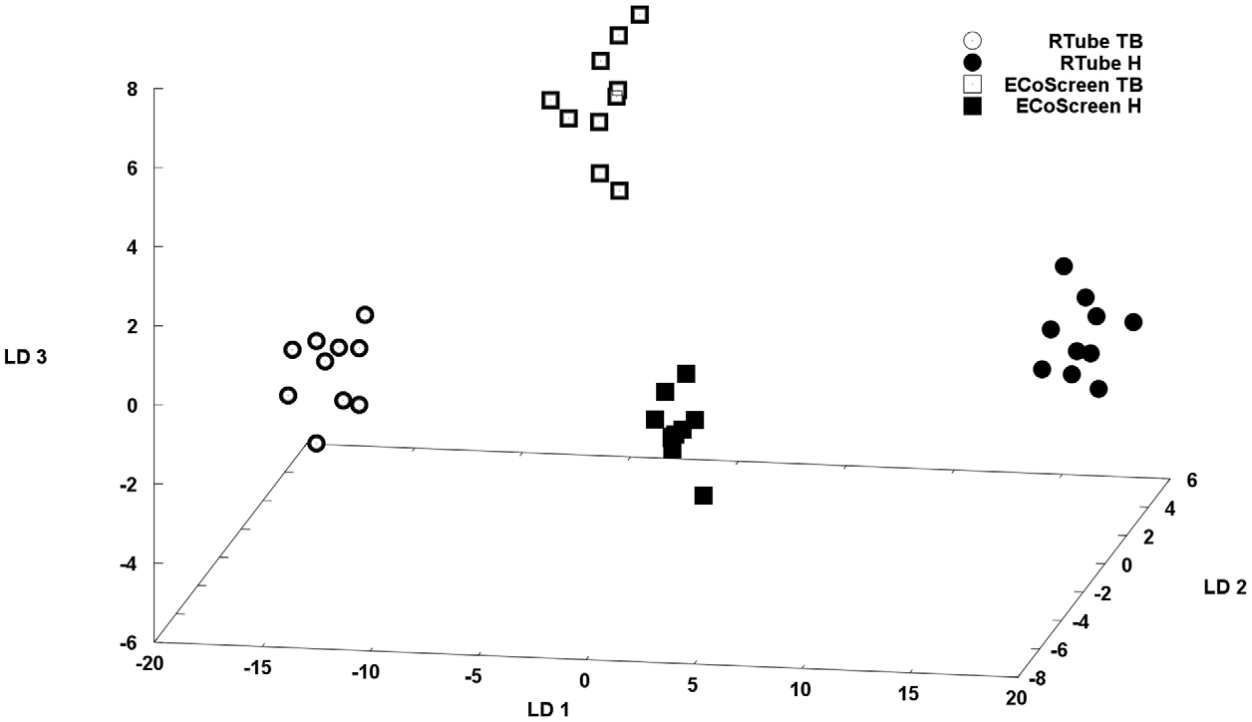
The three-dimensional plot of the linear discriminant (LD) analysis shows that two breathing patterns and the two devices were clearly separable using the electronic Nose (Cyranose 320).

The analysis of the VOC profiles of the four measurement series showed that both breathing patterns and the two devices were clearly separable. The Mahalanobis distances were all greater than 2, indicating good discriminative power. The cross-validation values (CVVs) were calculated with a cross-validation using ten percent of the training data as test data. The CVV reached values ranging from 64% to 87% (table 2, figure 6). Thus, different breathing maneuvers as well as the two used devices result in different VOC patterns and have to be standardized to ascertain repeatability of results.

**Table 2:** Mahalanobis distance and cross validation value (CVV; in parentheses).

	RTube H	ECoScreen TB	ECoScreen H
RTube TB	2.210	2.392	2.593
	(64%)	(74%)	(87%)
RTube H		2.551	2.227
		(79%)	(73%)
ECoScreen TB			2.378
			(74%)

The four groups (RTube or ECoScreen performing tidal breathing (TB) or hyperventilation (H)) were clearly distinguishable after analyzing the EBC with the electronic nose.

## Discussion

We have shown that the RTube device provided higher sample volumes compared to the ECoScreen Turbo. Furthermore, hyperventilation provided higher sample volumes compared to tidal breathing. Neither the device nor the breathing pattern influenced EBC pH. EBC conductivity remained relatively stable. Hyperventilation increased total protein amounts. The ECoScreen showed a trend towards higher total protein amounts. Neither SP-A or CCP concentrations were influenced by the breathing pattern. Normalization to the volume of EBC (absolute amount) or to the volume of ventilated air changed the results of the protein measurements dramatically. The electronic nose could distinguish between breathing pattern and device.

Interestingly, the RTube provided higher sample volumes compared to the ECoScreen Turbo. This is different from Soyer et al., who, using the EcoScreen 1, found that this device provided significantly higher sample volumes compared to the RTube [11]. One difference besides the assembly between the three devices is the cooling temperature during the sampling process. The ECoScreen I is constantly cooled to −10°C, and the ECoScreen Turbo, based on a modified wine cooler, has a constant cooling of approximately −4°C. The RTube has no constant cooling, but the temperature of the condenser usually starts at a fairly low temperature.

When comparing the RTube and the tested ECoScreen Turbo, the tube system length is another important difference in the assembly. The distance from the mouth to the collecting tube via a flexible tube system is much longer in the ECoScreen Turbo device compared to the RTube. It can be assumed that EBC volume is lost in the flexible tube system. Another possibility is a turbulent flow profile within the RTube which might increase the EBC volume, because there is more chance for a contact with the cooled walls of the collection tube.

We found no influence on EBC pH in comparing the breathing patterns suggesting that this parameter is robust. This confirms previously published data [15]. One might have expected a drop of the EBC pH after hyperventilation due to the possibility that hyperventilation might increase the amount of alveolar (and endobronchial) CO_2_, leading to an increased amount of H^+^ and HCO3^−^. However, we did not observe significant changes of the pH due to ventilatory manoeuvres.

Similar findings have been published regarding the comparisons of different devices [11], [18]. We also found no significant differences when comparing the RTube and ECoScreen Turbo regarding EBC pH. We extend the published comparisons to recently introduced devices. This is relevant, because the previously compared and used ECoScreen I cannot be purchased anymore and because pH in EBC has been proposed as a clinically relevant parameter to monitor airway inflammation in respiratory diseases, most often in asthma [19]–[21]. It is known that inflammation can change pH, but we ruled out systemic inflammation by clinical history and measures. Gastric reflux was also ruled out by clinical history, as it is also known to cause EBC acidification [22].

The hyperventilation maneuver tended to increase the total protein volumes in the EBC samples. Hyperventilation was monitored with an expiratory flow measure device provided by the manufacturer of the EBC device as described above and resulted in a 1.78-fold increased expiratory flow (data not shown). The increase of the total protein concentration did not seem not be caused by increased alveolar ventilation. We could show that SP-A, an alveolar marker, was not increased after hyperventilation. The bronchial marker CCP also showed no increased levels when performing hyperventilation. We performed ELISA and Western blots (data not shown) for CCP and SP-A, indicating no influence of the ventilation pattern on CCP or SP-A concentrations. To our knowledge, the total protein amount after performing hyperventilation has not been measured in EBC before. For mechanical ventilation in piglets it has been shown that hyperventilation increased albumin in the lavage [23]. Albumin measurements were not performed in our setup. One possible explanation for the trend of the higher protein concentrations might be shear stress forces. Protein volumes could also be higher because of the higher expiratory flow during hyperventilation, which might cause the blowing off of proteins from the bronchial branches, although this seems not very likely. Regarding the difference between the RTube and the ECoScreen Turbo, one might speculate that the RTube produces a more dilute sample because a similar volume of respiratory droplets is mixed with a greater volume of condensed water vapour as described above.

These results are also of interest when discussing different theories of about the source of exhaled respiratory droplets. It has been stated that respiratory tract turbulence results in the formation of aerosols out of the respiratory lining fluid [24]. However, Bondesson et al. conducted technetium-99 m studies in healthy subjects and concluded that EBC derives mainly from the central airways but that its composition of EBC would only partially reflect that of the epithelial lining fluid [25]. Moreover, Johnson and Morawska challenged the turbulence model and suggested an alternative model (bronchiole fluid film burst, BFFB) [24]. The proposed mechanism is based on a „process of respiratory fluid film or bubble bursting during the clearance of fluid closures which form in the lower bronchioles following exhalation”. The authors controlled the breathing pattern for inspiration and expiration separately. In contrast, we altered in- and expiration simultaneously by using voluntary „hyperventilation“. It might be that the primary mechanism involved the bursting menisci in bronchioles but that this was complemented by shear forces at higher ventilatory rates. As we did not see differences in SP-A or CCP we have to leave this question unanswered.

Different normalization processes are possible in EBC. One study tested (unsuccessfully) whether specific protein measurements could be normalized to the total protein amount to give more reliable results [26]. The problem arises from the presence of two carrier matrices, namely the EBC volume and the volume of ventilated air. Compared to measurements from fluids like blood, serum or urine, where the concentration of proteins depends on the absolute amount of protein and on the amount of only one carrier (fluid), the measurements of protein in EBC depend not only on the volume of EBC but also on the volume of ventilated air. This produces a high amount of complexity as shown in our figures 4 and 5 where the additional information of the volume of ventilated air changed the results dramatically. This is a very strong argument for either reporting ventilated volumes of air or using a standardized volume of ventilated air. We would advice to use a standardized volume of ventilated air to keep the “third parameter” (besides protein and fluid) constant.

By analyzing the EBC profile above the fluid surface of the samples we were able to show that the device and the breathing patterns caused a different VOC profile, which were clearly distinguishable with the electronic nose. This again strongly suggests that the breathing pattern should be standardized for healthy controls and probably also for patients, although they have not been tested in our setup. The results further showed that the device for sampling EBC did not seem to make a difference when analyzing robust parameters like EBC pH and (less so) EBC conductivity. For more sophisticated measurements such as protein measurements or pattern recognition performed with electronic noses, the device and the breathing pattern were of important influence. Our data indicate that device and breathing pattern cause sensible differences in the VOC composition. It is known that the expiratory resistance causes a difference in the VOC composition [27]. Other possible explanations include the cooling temperature and the above mentioned tube system in the ECoScreen Turbo which might be implicated by the loss of VOCs. The different breathing patterns had an influence of the VOCs which could have been caused by the above mentioned shear stress, resistance differences and a higher intrinsic positive end-expiratory pressure caused by dynamic hyperinflation which could be a reason for higher amounts of shear stress. This has been described as the case in a bench model of hyperventilation [28]. Further experiments should be performed to explore the cause of the differences in the VOCs caused by the devices and the breathing maneuvers.

Although we performed a carefully designed study there are some limitations. We only sampled healthy controls. The average age of the studied group was 24.8 years, reflecting a young age. Sampling a group of patients with obstructive lung disease might have strengthened the study. This should be performed in a fully powered experimental study, as the present one was a proof-of-concept study exploring important factors of influence. Regarding the RTube no expiration flow meter is commercially available; therefore the volume of ventilated air was assessed only using the ECoScreen Turbo. Further limitations arise from the purely cross-sectional nature of the study, as the results might differ over time.

In summary, EBC collection, analysis of pH and (partially) conductivity are extremely simple to perform, noninvasive, robust, inexpensive and comparable using the commercially available devices ECoScreen Turbo and RTube. The way of breathing seems not to have a major impact on these parameters. Therefore, it is well suited for noninvasive analysis in a longitudinal follow-up of individual patients, and patients can be provided with a portable device for collection of EBC at home.

Regarding the protein collection many questions remain unanswered. The breathing pattern seemed not to change the source of the EBC, since we could not show that CCP as marker for the bronchial fraction or SP-A as marker of the alveolar fraction were altered after hyperventilation. However, as the results of protein measurements, were greatly altered by the amount of ventilated air, the ventilated volume should be reported in further studies. For future measurements we recommend standardization of the amount of ventilated air (for example to 100 l) to gain better comparability between reports from different groups. This advice is further strengthened by the fact that using the C-320 it could be shown that the device and the breathing pattern had significant influence of the VOC pattern.

In conclusion we provide important results that increase the knowledge of the EBC sampling. Until now, EBC analysis has not entered clinical practice because of the lack of standardization of methods. Our results enhance the knowledge about the influence of the breathing pattern. Furthermore, we could show that ECoScreen Turbo and RTube display comparable values for robust, non-specific EBC markers like pH and (partially) conductivity. The total and specific protein values in EBC depend strongly on the underlying method of protein calculation and analysis. A comparison of protein markers in EBC will remain difficult with the current knowledge.
