# Correction: Comparison of Two Devices and Two Breathing Patterns for Exhaled Breath Condensate Sampling

**DOI:** 10.1371/journal.pone.0152620

**Published:** 2016-03-28

**Authors:** Eva-Maria Hüttmann, Timm Greulich, Janine Koepke, Christoph Nell, Akira Hattesohl, Severin Schmid, Sarah Noeske, Christian Herr, Gerrit John, Rudolf A. Jörres, Bernd Müller, Claus Vogelmeier, Andreas Rembert Koczulla

The authors would like to amend this article based on the discovery of a number of errors that came to light after publication.

We recognized a number of errors that made it necessary to repeat a part of the experiments. Please view the corrected text, table and figures here.

## 1. Study Population

We had to include a larger number of subjects, which changed the baseline characteristics of the study population. Therefore the paragraph “Study Population” under “Results” should read:

We included 43 healthy controls (22 male, 21 female) with a mean age of 25.56 years ± 4.22 years (18 years to 44 years), a mean BMI of 22.63 kg/m2 ± 3.02 kg/m2 (17.78 kg/m2 to 31.00 kg/m2). All were non-smokers and had no clinical signs of acute or chronic inflammation at the time of our measurements. As described above different subgroups of subjects participated in different parts of the study (setups). Details are displayed in the corrected Table 1 below.

**Table 1:** This table displays the participants’ characteristics that participated in different experimental setups. The number of individuals that participated in setups does not add up to the number of all participants, because some individuals participated in more than one experimental setup.

	n	Male/Female	Age [years]	BMI [kg/m^2^]
All Subjects	43	22/21	25.56 ± 4.22 (18–44)	22.63 ± 3.02 (17.78–31.00)
Setup I (Volume, pH, conductivity, eNose)	10	4/6	24.8 ± 2.78 (23–30)	21.52 ± 0.72 (18–26)
Setup II (Total protein)	10	7/3	25.8 ± 2.10 (24–29)	22.0 ± 4.01 (17.8–27.8)
Setup III (CCP, SP-A)	12	5/7	26.08 ± 3.63 (20–31)	21.71 ± 2.34 (17.78–24.64)
Setup IV (AAT in uncoated collection tubes)	10	6/4	28.8 ± 5.83 (24–44)	24.0 ± 4.40 (18–28)
Setup V (AAT in coated collection tubes)	12	5/7	23.7 ± 2.93 (18–25)	22.0 ± 1.83 (18.8–24.9)

AAT: alpha-1- antitrypsin; CCP: clara cell secretory protein; SP-A: surfactant protein A

For our experiments EBC was collected from healthy non smoking controls (n = 43, 25.56 years ± 4.22 years, BMI 22.63 kg/m2 ± 3.02 kg/m2, 22 male / 21 female) that showed no clinical signs of inflammation at the time of their measurement. All participants underwent a single study visit during which EBC was collected four times in a crossover design, twice with every device; once with every device while performing hyperventilation and once with tidal breathing.

For analysis of EBC pH, conductivity and the electronic nose derived measurements, EBC was collected from 10 healthy non-smoking controls at the age of 24.8 years ± 2.78 years (23 years to 30 years). To analyze total protein amount a second set of ten test persons at the age of 25.8 years ± 2.1 years (24 years to 29 years) performed the same maneuvers as described above. In a third set of 12 subjects at the age of 26.08 years ± 3.63 years (20 years to 31 years), CCP and SP-A were measured using coated collection tubes, after using uncoated tubes had not yielded high enough results. In a fourth set of 10 subjects at the age of 28.8 years ± 5.83 years (24 years to 44 years) we measured AAT in uncoated collection tubes. Finally, in a fifth setup, 12 healthy non smoking controls at the age of 23.7 years ± 2.93 years (18 years to 25 years) performed the same experiments to compare the amount of AAT collected in coated collection tubes with uncoated collections tubes.

## 2. Total Protein Concentration

Originally, we measured total protein concentration in exhaled breath condensate using a NanoDrop 1000 (PeqLab Biotechnologie GmbH, Erlangen, Germany) that had a detection limit of 0.1 mg/ml, but the values displayed in the manuscript were below that limit. We corrected theses analyses (Subjects from setup II in Table 1):

Total protein concentration was measured using a commercial available kit for bicinchoninic acid (Quanti Pro Tm BCA Assay Kit, Sigma Aldrich, Germany). The linear range of the essay was 0.5 μg/ml– 30 μg/ml. For BCA Assay the whole sample of EBC was lyophilizied by using a speed vac (Bachofer, Reutlingen, Germany) and the sample was resuspended in a volume of 20 μl. Absorption at 562 nm was detected using a Tecan Ultra 384 Reader (TECAN Infinite^®^ 200 PRO, Crailsheim, Germany).

Comparing the two different devices performing tidal breathing manoeuvres the ECoScreen yielded higher protein concentrations in EBC than the RTube (ECoScreen: 1.725 μg/ml ± 0.695 μg/ml vs. RTube: 1.470 μg/ml ± 1.227 μg/ml; p = 0.56 revised Fig 4a, left two bars), although the difference was not statistically significant. When performing hyperventilation the ECoScreen resulted also in higher protein concentrations than the RTube but not statistically significant (ECoScreen: 2.617 μg/ml ± 1.219 μg/ml vs. RTube: 1.827 μg/ml ± 1.199 μg/ml; p = 0.23; revised Fig 4a, right two bars).

**Fig 4:**
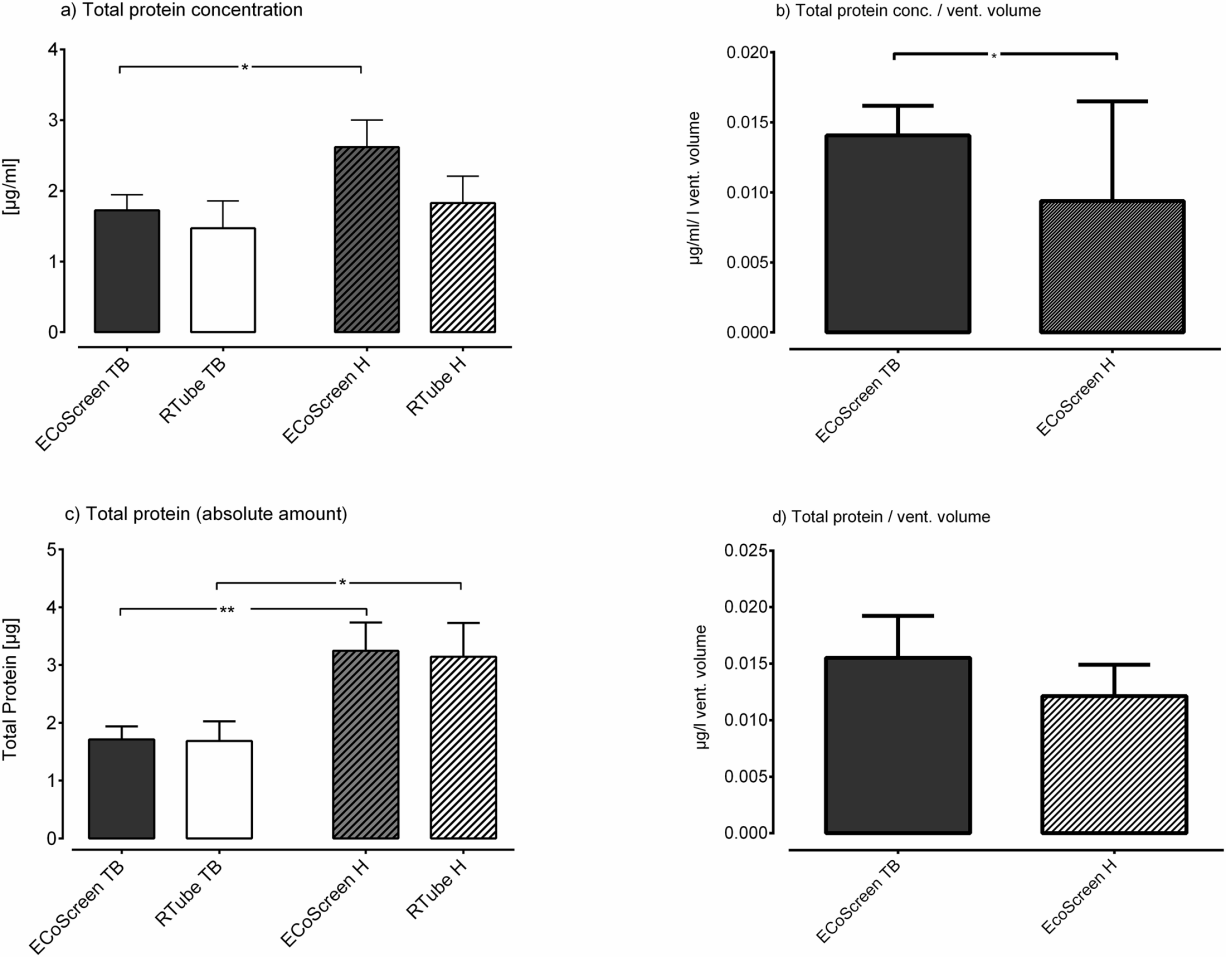
Displayed are the overall protein measurements in four different ways. a) Comparing the ECoScreen and RTube EBC protein concentration after tidal breathing (TB) and hyperventilation (H) no statistical significant difference could be shown. Comparing the two manoeuvres, hyperventilation yielded higher concentrations than tidal breathing, but this difference was significant only in the ECoScreen (p < 0.05). b) When the overall protein concentrations were normalized to the volume of ventilated air, tidal breathing yielded higher normalized concentrations than hyperventilation (p < 0.05). c) The device had no influence on the total protein amount. Within the same device, hyperventilation yielded higher absolute amounts than tidal breathing (RTube: p < 0.05; ECoScreen: p < 0.005). d) By normalizing the absolute protein amount in EBC to the volume of ventilated air we did not find a significant difference between tidal breathing and hyperventilation.

Within the same device hyperventilation yielded higher overall protein concentrations of EBC than tidal breathing, though the difference was statistically significant only in the ECoScreen device (H: 2.617 μg/ml ± 1.219 μg/ml vs. TB: 1.715 μg/ml ± 0.695 μg/ml; p < 0.05; revised Fig 4a, grey bars).

When the overall protein concentrations were normalized to the volume of ventilated air (ECoScreen Turbo only), tidal breathing yielded higher normalized concentrations than hyperventilation (TB: 0.0141 μg/ml/l ± 0.0067 μg/ml/l; H: 0.0094 μg/ml/l ± 0.0071 μg/ml/l; p < 0.05; revised Fig 4b).

When we plotted the total protein amount instead of the protein concentration (thus, we multiplied the concentration with the volume of obtained EBC), the results remained rather stable. The device had no influence on the total protein amount, neither in tidal breathing (ECoScreen: 1.713 μg ± 0.7129 μg vs. RTube: 1.685 μg ± 1.084 μg; p = 0.95; revised Fig 4c, left bars), nor in hyperventilation (ECoScreen: 3.246 μg ± 1.545 μg vs. RTube: 3.142 μg ± 1.85 μg; p = 0.89; revised Fig 4c, right bars). Within the same device, hyperventilation yielded higher absolute amounts than tidal breathing. The difference was significant for the RTube (H: 3.142 μg ± 1.85 μg vs. TB: 1.685 μg ± 1.084; p < 0.05; revised Fig 4c, white bars) as well as for the ECoScreen (H: 3.246 μg ± 1.545 μg vs. TB: 1.713 μg ± 0.7129; p < 0.005; revised Fig 4c, grey bars).

Finally, we normalized the absolute amount to the volume of ventilated air (ECoScreen Turbo only). With this method we did not find significant differences (H: 0.0121 μg/l ± 0.0088 μg/l vs. TB: 0.0155 μg/l ± 0.0117 μg/l; p = 0.13; revised Fig 4d).

## 3. Specific Protein Measurements

Because the setting of the TECAN Ultra 384 reader used for the ELISA of Clara cell secretory protein (CCP) and surfactant protein-A (SP-A) were incorrect, these experiments had to be repeated, too (subjects from setup III in Table 1).

More than 50% of measurements regarding CCP and SPA were below the limit of detection. Precoating the collection tubes with 1% BSA (bovine serum albumin) in PBS and concentrating the sample (following lyophilization) did not yield consistent results within the linear range of detection (setup III).

We continued the approach of specific protein measurements choosing alpha-1-antitrypsin (AAT) based on previous results using the same methods [Koczulla AR, Noeske S, Herr C, Koepke J, Jorres RA, Nell C, Schmid S, Vogelmeier C, Bals R. Alpha-1 antitrypsin is elevated in exhaled breath condensate and serum in exacerbated COPD patients. Respir Med 2012 Jan; 106: 120–126].

To evaluate the influence of the collection system on specific proteins, we performed one setup with uncoated collection tubes (setup IV) and one setup with coated collection tubes (setup V).

AAT (as has been demonstrated before) was consistently detectable in EBC (collected in uncoated–setup IV—and coated collection tubes–setup V). The concentration of detectable AAT was significantly higher in precoated tubes compared to uncoated tubes (factor 3.6 regarding in tidal breathing, factor 1.79 in hyperventilation; data not shown). For further experiments, the results for coated collections tubes are reported: Regardless of the way the results were reported (concentration, total amount, normalization to ventilated volume), the values for AAT derived through hyperventilation were consistently higher than the values derived through tidal breathing. This was significant for the concentration (H: 2439 pg/ml ± 1358 pg/ml vs. TB: 1056 ± 759.9 pg/ml; p < 0.005; revised Fig 5a), the amount (H: 3608 pg ± 2724 pg vs. TB: 1159 pg ± 864.3 pg; p < 0.005; revised Fig 5c), the normalized amount (H: 13.43 pg/l ± 10.38 pg/l vs. TB: 6.643 pg/l ± 4.908 pg/l; p < 0.05; revised Fig 5d), and close to significant for the normalized concentration (H: 9.056 pg/ml/l ± 5.3 pg/ml/l vs. TB: 6.148 pg/ml/l ± 4.523 pg/ml/l; p = 0.08; revised Fig 5b).

**Fig 5:**
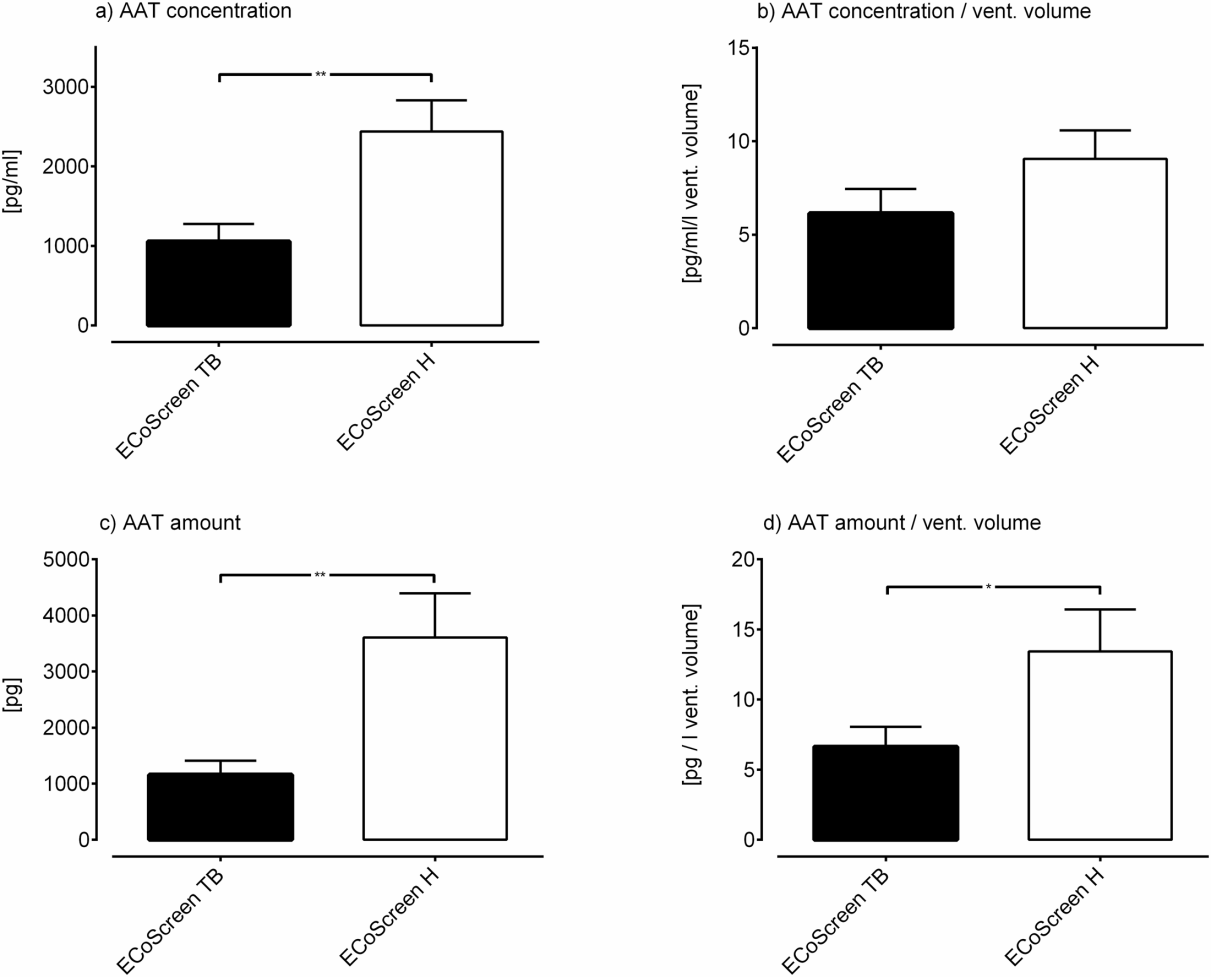
AAT measurements are displayed in four different ways. Regardless of the way the results were reported, the values for AAT derived through hyperventilation were consistently higher than the values derived through tidal breathing: a) concentration (p < 0.005); b) normalized concentration (p = 0.08) c) total amount (p < 0.005); d) normalized amount (p < 0.05).

## 4. Changes to the discussion

a) In the first paragraph of the discussion, we stated that SP-A and CCP were not influenced by the breathing pattern. As pointed out, these specific proteins were not consistently detectable in EBC. Therefore, the first paragraph should read:

We have shown that the RTube device provided higher sample volumes compared to the ECoScreen Turbo. Furthermore, hyperventilation provided higher sample volumes compared to tidal breathing. Neither the device nor the breathing pattern influenced EBC pH. EBC conductivity remained relatively stable. Hyperventilation increased total protein amounts. The ECoScreen showed a trend towards higher total protein amounts. SP-A and CCP were not consistently detectable in EBC. Normalization to the volume of EBC (absolute amount) or to the volume of ventilated air changed the results of the protein measurements in a relevant manner. The electronic nose could distinguish head space volatiles of EBC between breathing pattern and device.

b) In sixth and seventh paragraph of the Discussion, we discussed the origin of proteins in EBC. Since SP-A and CCP data were not consistent, the corrected paragraphs read:

The hyperventilation maneuver tended to increase the total protein volumes, and the AAT volumes in the EBC samples. Hyperventilation was monitored with an expiratory flow measure device provided by the manufacturer of the EBC device as described above and resulted in a 1.78-fold increased expiratory flow (data not shown). To our knowledge, neither the total protein amount nor the AAT amount after performing hyperventilation has been measured in EBC before. For mechanical ventilation in piglets it has been shown that hyperventilation increased albumin in the lavage [24]. Albumin measurements were not performed in our setup. One possible explanation for the trend of the higher protein concentrations might be shear stress forces. Protein volumes could also be higher because of the higher expiratory flow during hyperventilation, which might cause the blowing off of proteins from the bronchial branches. Regarding the difference between the RTube and the ECoScreen Turbo, one might speculate that the RTube produces a more dilute sample because a similar volume of respiratory droplets is mixed with a greater volume of condensed water vapour as described above.

These results are also of interest when discussing different theories of about the source of exhaled respiratory droplets. It has been stated that respiratory tract turbulence results in the formation of aerosols out of the respiratory lining fluid [25]. However, Bondesson et al. conducted technetium-99 m studies in healthy subjects and concluded that EBC derives mainly from the central airways and that the composition of EBC would only partially reflect that of the epithelial lining fluid [26]. Moreover, Johnson and Morawska challenged the turbulence model and suggested an alternative model (bronchiole fluid film burst, BFFB) [25]. The proposed mechanism is based on a “process of respiratory fluid film or bubble bursting during the clearance of fluid closures which form in the lower bronchioles following exhalation”. The authors controlled the breathing pattern for inspiration and expiration separately. In contrast, we altered in- and expiration simultaneously by using “hyperventilation”. It might be that the primary mechanism involved the bursting menisci in bronchioles but that this was complemented by shear forces at higher ventilatory rates. Because we were not able to detect SP-A or CCP consistently, we have to leave this question unanswered.

c) In the penultimate paragraph of the Discussion, the second sentence relates again to SP-A and CCP and is incorrect. This paragraph should read:

Regarding the protein collection many questions remain unanswered. However, as the results of protein measurements, were greatly altered by the amount of ventilated air, the ventilated volume should be reported in further studies. For future measurements we recommend standardization of the amount of ventilated air (for example to 100 l) to gain better comparability between reports from different groups. This advice is further strengthened by the fact that using the C-320 it could be shown that the device and the breathing pattern had significant influence of the VOC pattern.

## 5. Changes to the abstract

a) In the introduction of the abstract we wrote that we aimed to analyze the source of EBC. Because SP-A and CCP were not consistently measurable, this sentence is incorrect. The paragraph should read:

Analysis of exhaled breath condensate (EBC) is a non-invasive method to access the epithelial lining fluid of the lungs. Due to standardization problems the method has not entered clinical practice. The aim of the study was to assess the comparability for two commercially available devices in healthy controls. In addition, we assessed the influence of different breathing patterns in healthy controls on total protein amount and specific proteins in EBC.

b) Under “Methods” we stated that we collected EBC in ten subjects. As stated above we had to include 43 subjects to repeat and to complete all experiments. Furthermore, we measured AAT in EBC. Therefore, the paragraph “Methods” should read:

EBC was collected in different experimental setup (≥ 10 individuals each) using the RTube and ECoScreen Turbo in a randomized crossover design, twice with every device—once in tidal breathing and once in hyperventilation. EBC conductivity, pH, surfactant protein A, Clara cell secretory protein, alpha-1-antitrypsin and total protein were assessed. Bland-Altman plots were constructed to display the influence of different devices or breathing patterns and the intra-class correlation coefficient (ICC) was calculated. The volatile organic compound profile was measured using the electronic nose Cyranose 320. For the analysis of these data, the linear discriminant analysis, the Mahalanobis distances and the cross-validation values (CVV) were calculated.

The authors would like to apologize for these errors and confirm that these corrections do not affect the conclusions presented in the article. However, they do affect parts of the abstract.
